# Mapping the Architecture of Ferret Brains at Single-Cell Resolution

**DOI:** 10.3389/fnins.2020.00322

**Published:** 2020-04-15

**Authors:** Ben Long, Tao Jiang, Jianmin Zhang, Siqi Chen, Xueyan Jia, Xiaofeng Xu, Qingming Luo, Hui Gong, Anan Li, Xiangning Li

**Affiliations:** ^1^Britton Chance Center for Biomedical Photonics, Wuhan National Laboratory for Optoelectronics-Huazhong University of Science and Technology, Wuhan, China; ^2^MoE Key Laboratory for Biomedical Photonics, School of Engineering Sciences, Huazhong University of Science and Technology, Wuhan, China; ^3^HUST-Suzhou Institute for Brainsmatics, JITRI Institute for Brainsmatics, Suzhou, China

**Keywords:** cytoarchitectonics, en-bloc Nissl staining, whole-brain imaging, single-cell resolution, giant pyramidal neuron, ferret brains

## Abstract

Mapping the cytoarchitecture of the whole brain can reveal the organizational logic of neural systems. However, this remains a significant challenge, especially for gyrencephalic brains with a large volume. Here we propose an integrated pipeline for generating a cytoarchitectonic atlas with single-cell resolution of the whole brain. To analyze a large-volume brain, we used a modified en-bloc Nissl staining protocol to achieve uniform staining of large-scale brain specimens from ferret (*Mustela putorius furo*). By combining whole-brain imaging and big data processing, we established strategies for parsing cytoarchitectural information at a voxel resolution of 0.33 μm × 0.33 μm × 1 μm and terabyte-scale data analysis. Using the cytoarchitectonic datasets for adult ferret brain, we identified giant pyramidal neurons in ferret brains and provide the first report of their morphological diversity, neurochemical phenotype, and distribution patterns in the whole brain in three dimensions. This pipeline will facilitate studies on the organization and development of the mammalian brains, from that of rodents to the gyrencephalic brains of ferret and even primates.

## Introduction

Mapping the cytoarchitecture of the vertebrate brains can provide insight into its organizational logic and functions (Dong, 2008; Swanson, 2012). Recent advances in tissue processing and volumetric imaging have revealed the three-dimensional (3D) structure of cells and organs (Richardson and Lichtman, 2015; Susaki and Ueda, 2016). However, cytoarchitectonic analysis of intact brains remains a significant challenge, especially for the large-volume brains.

Nissl staining is the most widely used method for cytoarchitectonic and morphometric studies as it allows visualization of cell bodies and proximal dendrites (Van De Werd et al., 2010; Wu et al., 2014), thereby providing information on the size, shape, orientation, and density of cells in tissues, which is useful for developing brain reference atlases and identifying landmarks in the neural circuits (Dong, 2008; Erö et al., 2018). Fluorescent dyes such as SYTO and Hoechst stains mainly label cell nuclei while Nissl bodies are weakly stained (Susaki et al., 2014; Seiriki et al., 2017), precluding the delineation of soma shape and orientation (Niu et al., 2015; Chang and Kawai, 2018) that is critical for distinguishing brain regions. Moreover, Nissl staining is a slow and complex process and its application is thus limited to thin tissue sections or tissue blocks. We previously reconstructed the cytoarchitecture of mouse brain by combining a modified Nissl staining protocol with whole-brain imaging techniques (Wu et al., 2014, 2016; Xiong et al., 2017). NeuN-staining also can be used to perform stereological analysis of almost all neurons in neural systems but depends heavily on the quality of immunostaining for sections or en-bloc brain tissues and time-consuming (Herculano-Houzel and Lent, 2005; Atapour et al., 2019; Yun et al., 2019). There is a growing trend of studying brain structure in 3D in primates and human instead of rodents (Richardson and Lichtman, 2015; Susaki and Ueda, 2016). However, the expanded volume caused by the presence of gyri and sulci limits the analysis of larger and more complex brains (DeFelipe, 2015) in terms of labeling, imaging, and data analysis (Yuan et al., 2015). As such, there is a need for more suitable approaches for analyzing whole-brain cytoarchitecture in mammals.

To date, several cytoarchitectonic atlases of large brains have been established with the aid of magnetic resonance imaging for stereotaxic coordinates, including for tree shrew (*Tupaia belangeri chinensis*) (Zhou and Ni, 2016), ferret (*Mustela putorius furo*) (Radtke-Schuller, 2018), common marmoset (*Callithrix jacchus*) (Yuasa et al., 2010; Paxinos et al., 2012; Liu et al., 2018), and rhesus monkey (*Macaca mulatta*) (Paxinos et al., 2008; Saleem and Logothetis, 2012; Reveley et al., 2017). However, the low-resolution data thus obtained, especially during continuous 3D scanning and analysis, do not reveal fine structures. At a basic level, obtaining accurate cytoarchitectonic information of the whole brain requires uniform and en-bloc staining of tissue samples.

In this study we developed an integrated pipeline for whole-brain cytoarchitectonic atlas construction that combines a modified protocol for large-volume en-bloc Nissl staining with whole-brain imaging techniques (Li et al., 2010; Wu et al., 2014), data processing methods developed in our earlier work (Quan et al., 2013; Li et al., 2017), and a newly trained 3D U-Net deep learning model (Ronneberger et al., 2015; Çiçek et al., 2016; Dong et al., 2018) for acquisition and analysis of a whole brain dataset. This strategy enabled high-quality uniform cell staining, visualization of somata throughout the brain, and segmentation of brain structures at single-cell resolution. We applied this pipeline to ferret and identified for the first time giant pyramidal neurons (PyNs) in the ferret brain.

## Materials and Methods

### Experimental Model

Animal experiments were approved by the Institutional Animal Ethics Committee of Huazhong University of Science and Technology. Three adult female experimental ferrets (2 years old; Wuxi Sangosho Biotech. Co., Wuxi, China) were used for experiments, among them two ferrets were used for en-bloc Nissl staining and whole-brain imaging and one was used for immunostaining. All animals were maintained at 20°C ± 2°C on a 12:12-h light/dark cycle, with free access to food and water.

### Tissue Preparation

After a lethal dose of anesthetic, the animals were transcardially perfused with 0.01 M phosphate-buffered saline (PBS) (Sigma-Aldrich, St. Louis, MO, United States; P3813) and 4% paraformaldehyde (PFA) (Sigma-Aldrich; 158127) using a peristaltic pump. The intact brains were dissected and post-fixed in 4% PFA (containing 0.05% sodium azide) at 4°C for 4 days. The brains were then rinsed for 2 days at 4°C in 0.01 M PBS and prepared for large-volume en-bloc Nissl staining and immunolabeling.

### Large-Volume En-Bloc Nissl Staining

En-bloc Nissl staining of the intact two female ferret brains were performed as previously described (Wu et al., 2014, 2016). Briefly, the brains were immersed in 2.5% thionine solution with gentle shaking for 60 days at room temperature. After Nissl staining, the brains were transferred to 70% ethanol (w/w) for 30 days to wash out excess stain, with the 70% ethanol replaced every 12 h. The thionine solution was prepared by dissolving 2.5 g thionine acetate salt (Sigma-Aldrich; 88930) in freshly prepared 100 ml acetic acid-sodium acetate buffer solution (pH 5.0) with constant stirring for 2 days, and then passed through filter paper and stored at room temperature.

The Spurr resin embedding procedure has been previously described (Wu et al., 2014) and was used in the present study with modifications to the dehydration, resin infiltration, and polymerization steps. The Nissl-stained ferret brains were dehydrated in a graded series of ethanol and acetone solutions (85, 95, and 100% ethanol; 1:1 ethanol/acetone mixture; and 100% acetone for 12 h each, followed by and 100% acetone for 24 h). The brains were then infiltrated in a graded series of Spurr resin solutions (50, 75, and 100% resin solution for 1 day each, followed by 100% resin solution for 4 days with replacement of the solution every 2 days). The brains were placed in a rectangular plastic mold filled with fresh 100% Spurr resin solution and the orientation was adjusted during the polymerization process to optimize the sectioning angle, which was followed by polymerization for 2 days at 60°C. Importantly, the resin solution was freshly prepared and the 50 and 75% resin solutions were diluted in 100% acetone.

### Histology and Imaging

To characterize the neurochemical phenotypes of giant pyramidal neurons in ferret brains another female ferret brain was used for immunolabeling. The brain was sectioned at a thickness of 70 μm on a vibratome (VT 1200S; Leica, Wetzlar, Germany) and sections through the motor cortex of the posterior sigmoid gyrus were selected for histological analysis.

The sections were washed three times for 10 min in PBS, permeabilized with PBS with 0.3% Triton-X-100 in PBS (PBST) for 1 h, and then incubated with blocking solution (5% bovine serum albumin in PBST) for 1 h followed by primary antibodies for 24 h at 4°C. The following primary antibodies were used: rabbit anti-neuronal nuclei (NeuN) (1:800 dilution; Abcam, Cambridge, MA, United States; ab177487); goat anti-excitatory amino acid transporter (EAAC)1 (1:1000 dilution; Millipore, Billerica, MA, United States; AB1520); mouse anti-glutamic acid decarboxylase (GAD)67 (1:1,000 dilution; Millipore, MAB5406); and mouse anti- parvalbumin (PV) (1:1,000 dilution; Millipore, MAB1572). After rinsing with PBS, the sections were incubated overnight at 4°C with appropriate fluorophore-conjugated secondary antibody followed by three 10-min washes in PBS. The following secondary antibodies were used: Alexa Fluor 594-conjugated donkey anti-rabbit immunoglobulin G (IgG) and goat anti-rabbit IgG (A-21207 and A-11037, respectively); and Alexa Fluor 488-conjugated donkey anti-goat IgG and goat anti-mouse IgG (A-11055 and A-11001, respectively). All were used at 1:800 dilution and were obtained from Invitrogen (Carlsbad, CA, United States). The sections were stained with blue fluorescent Nissl dye (1:500 dilution; Invitrogen; N21479) in PBS for 10 min and washed once with PBS prior to mounting with FluoroGel. The sections were imaged with a Leica SP8 confocal microscope (20×, 0.75 NA) and processed using ImageJ software (National Institutes of Health, Bethesda, MD, United States).

### Whole-Brain Imaging and Image Preprocessing

Whole-brain imaging and image preprocessing were performed on two female intact Nissl-stained resin-embedded ferret brains. The brains were placed in a Micro-Optical Sectioning Tomography (MOST) system (BioMapping 1000; Wuhan OE-Bio Co., Wuhan, China) for automatic whole-brain imaging (Li et al., 2010). The system was composed of a microtome, light microscope, and imaging recorder module that simultaneously performed thin sectioning and image scanning. The specimen was cut into strips with a diamond knife and immediately imaged by reflected bright-field microscopy, guided by a motorized precision 3D (XYZ) stage that extended the imaging area by 1-μm Z steps. Continuous whole-brain imaging lasted 64 days at a voxel resolution of 0.33 μm × 0.33 μm × 1 μm (40×, 0.8 NA objective). The images were saved as 8-bit grayscale images; the uncompressed image dataset comprised more than 11,000 horizontal sections and exceeded 22.0 terabytes.

The original images were preprocessed as previously described (Ding et al., 2013; Wu et al., 2014). Briefly, the images were stitched together, and periodic noise was removed and non-uniform illumination was calibrated using by mean projection curve in a customized program, so that the coronal or sagittal sections had a uniform intensity (Ding et al., 2013; Wu et al., 2014). The raw dataset was converted to TDat format for petabyte-scale data calculation including re-slicing, maximum intensity projection, and giant PyNs localization (Li et al., 2017), which split the whole-brain dataset into a series of 512 μm × 512 μm × 512 μm blocks in the data space. Pre-processing was performed on a computing server (72 cores, 2 GHz/core) and graphical workstation (Dell, Round Rock, TX, United States; T7920) and took about 1 day.

### K-Means Clustering

To analyze the features of the giant PyNs soma five data blocks in the ferret motor cortex at a voxel size of 300 μm × 300 μm × 300 μm were randomly selected, and all stained cells in these blocks were manually segmented using the segmentation editor module of Amira v6.1.1 software (FEI, Villebon sur Yvette, France); the volume, mean gray value, and longest radius of each cell were then calculated.

To analyze differences in cell volume between giant PyNs and other PyNs the two cell types were manually identified according to previously defined criteria (White et al., 1997; Rivara et al., 2003). To further analyze giant PyNs K-means clustering, an unsupervised machine learning technique, was performed for all cells with cell volume, mean gray value, and longest radius as the three principal components (Xu and Tian, 2015; Arora et al., 2016). The cluster centroids representing the center of data points of giant PyNs and other cells were iteratively updated until objects in the same cluster showed high similarity while those in different clusters showed lower similarity.

### Localization of Giant Pyramidal Neurons in Whole Ferret Brains

A high-throughput processing scheme was used for brain-wide localization and segmentation of giant PyNs. NeuroGPS software (Quan et al., 2013) was used to locate the somata of the cells in one of the whole ferret brain; 3D data blocks were extracted based on the corresponding 3D coordinates of the center of the soma, and the soma was automatically segmented using the 3D U-Net model (Çiçek et al., 2016).

Automatic localization and quantification of giant pyramidal neurons were performed with the NeuroGPS algorithm (Quan et al., 2013). 3D data blocks (128 μm × 128 μm × 128 μm) containing giant pyramidal neurons were cropped one at a time from the ferret brain datasets based on 3D coordinates. To assess the accuracy of automatic localization, we compared the results obtained by automatic and manual methods. The recall value was defined as co-detected total cells by two-approach/manually detected total cells, and the precision value was defined as co-detected total cells by two-approach/automatically detected total cells (He et al., 2014). We randomly selected five data blocks with a voxel size of 512 μm × 512 μm × 512 μm containing giant PyNs and calculated the recall and precision values. Three skilled investigators verified the automatic localization results.

### Soma Segmentation of Giant Pyramidal Neurons

For volumetric soma segmentation of giant PyNs, we trained a deep learning model to automatically segment the somata of giant PyNs with a 3D U-Net-based convolutional neural network (Çiçek et al., 2016). A data augmentation strategy was used to improve the efficiency of network training (Ronneberger et al., 2015), and manually segmented giant PyNs (*n* = 286 cells) were used to train the 3D U-Net model. Surface rendering was performed using Imaris v.9.0, software (Bitplane, Zurich, Switzerland). Automatically segmented somata were quantitated using a customized algorithm. The parameters of Betz cell somata including Max_section distance ratio, Rectangle axial ratio, and Max_section axial ratio.

### 3D Visualization

For rendering of data blocks and giant PyNs somata, we used the surface tools in Imaris software. For visualization of whole ferret brain datasets, we used Amira software to generate figures of volume or surface rendering. The 3D coordinates of giant PyNs were transformed into SWC format and rendered in whole ferret brain datasets with a resampled voxel resolution of 20 μm × 20 μm × 20 μm. All giant PyNs or giant PyNs with four distinct soma shapes as well as the outline of the whole brain were simultaneously loaded in Amira software to generate figures of whole-brain volume rendering.

### Quantification and Statistical Analysis

All violin plots and graphs were generated using Prism v8.0 software (GraphPad, La Jolla, CA, United States). Data were analyzed with the two-tailed Student’s *t* test or by one-way analysis of variance followed by Tukey’s *post hoc* test using Prism and SPSS v22 software (IBM SPSS Statistics, Armonk, NY, United States). The confidence level (*P* value) was set to 0.05 and results are presented as mean ± standard error of the mean.

## Results

### Pipeline for Establishing a Whole-Brain Cytoarchitectonic Atlas of Large-Scale Brains

**FIGURE 1:**
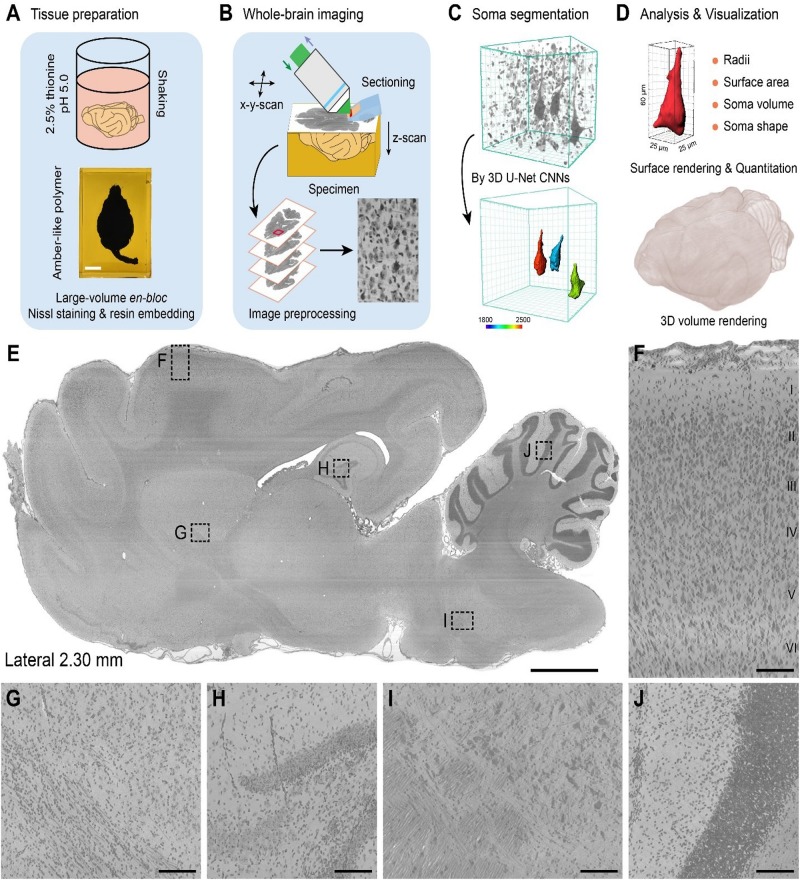
Pipeline for the construction of a whole-brain cytoarchitectonic atlas of large-scale brains. **(A)** Large-volume en-bloc Nissl staining and resin embedding procedures for intact ferret brains. Scale bar, 1 cm. **(B)** Whole-brain imaging and image preprocessing by MOST system. **(C)** 3D soma segmentation by 3D U-Net convolution neural network (CNN). Giant PyNs were identified and segmented using trained 3D U-Net CNNs and surface rendering was performed with Imaris software with a colored bar from a surface area of 1800–2500 μm^2^ and 3D data block of 128 μm × 128 μm × 128 μm. **(D)** Quantitation and 3D visualization of automatically segmented somata or whole brain. **(E)** Representative en-bloc Nissl-stained sagittal plane result, locating in the right hemisphere about 2.30 mm from midline to lateral side (20 μm thickness). Scale bar, 2 mm. **(F)** Laminar cytoarchitecture in the neocortex of ferret brains. Layer I to VI can be clearly distinguished. Enlarge view from dotted box in **(E)**. Scale bar, 100 μm. **(G–J)** Enlarged images from boxes in **(E)** showed uniform staining throughout the entire brain. Scale bar, 100 μm.

The workflow for the cytoarchitectonic atlas had four components: (i) large-volume en-bloc Nissl staining, (ii) whole-brain imaging, (iii) soma segmentation, and (iv) data analysis and visualization (Figures 1A–D).

For large-volume en-bloc Nissl staining, the post-fixed intact brains were immersed in a slightly acidic (pH 5.0) solution of 2.5% thionine with gentle shaking, followed by rinsing with 70% ethanol (Figure 1A and Supplementary Figures S1A–C). Given the speed of penetration of the solution into the tissue, the staining time and washing period was extended for the large-volume tissue samples. Thus, the intact ferret brains, with a volume over 6 cm^3^, were maintained in the acidic thionine solution for over 60 days to ensure full tissue penetration. Subsequent rinsing in 70% ethanol for over 30 days prevented excessive staining and improved the signal contrast between stained cells and surrounding tissue. The constant environment, gentle shaking, and long duration of continuous staining and rinsing ensured adequate, uniform staining for the large-volume tissues (Wu et al., 2014, 2016).

To enable mechanical sectioning at the micron level during high-resolution whole-brain imaging (Li et al., 2010; Wu et al., 2014), the tissues were embedded in resin. After sufficient dehydration in a graded ethanol/acetone series, the intact brains were infiltrated in a graded series of Spurr resin, followed by thermal polymerization (Supplementary Figures S1C–F). This procedure of en-bloc Nissl staining and resin embedding preserved the quality of the brain tissues for whole-brain imaging. The length of each step can be adjusted according to size or volume of the brain being examined.

Whole-brain imaging was performed with the MOST system (Figure 1B). With the moving 3D translation stage harboring the sample, strips were imaged in 1-μm Z steps on the rake face of the diamond knife by reflected bright-field microscopy, which continued without interruption until a dataset for the whole brain was acquired (Li et al., 2010). The strip images were then preprocessed by tile stitching and illumination correction using a customized program (Ding et al., 2013; Wu et al., 2014). The whole-brain imaging enabled the extraction of a large-scale dataset at a voxel resolution of 0.33 μm × 0.33 μm × 1 μm. The raw dataset was converted into TDat format for processing (re-slicing, brain-wide soma localization, etc.) of a terabyte-sized whole-brain dataset and for calculations (Li et al., 2017). The whole ferret brain dataset exceeded 22.0 terabytes and comprised more than 11,000 horizontal sections, providing single-cell-resolution images that could be used to identify features of individual cells.

To analyze the distribution patterns of somata, individual cells were located using the NeuroGPS algorithm (Quan et al., 2013) and automatic soma segmentation was performed using a newly trained 3D U-Net deep learning model (Ronneberger et al., 2015; Çiçek et al., 2016; Dong et al., 2018). A subpopulation of cells was well segmented (Figure 1C). Their somata were visualized and quantified using Imaris and Amira software programs with a customized algorithm and high-throughput data processing scheme (Figure 1D).

Finally, we selected representative sagittal plane near midline (Figure 1E) and enlarged high-magnification images (Figures 1F–J) to systematically evaluate the effect of en-bloc Nissl staining with whole-sample continuous imaging. These results indicated that the whole ferret brain datasets were uniform staining with high-contrast, high-resolution with the aid of integration of modified large-volume en-bloc Nissl staining and whole-brain imaging. As shown in Figure 1F, the laminar cytoarchitecture in the ferret neocortex (layer I to VI) could be clearly distinguished. Our cytoarchitectonic atlas workflow enables uniform cytoarchitectonic staining, whole-brain imaging, automatic soma segmentation and quantization of various cells of interest at single-cell resolution in large-scale brains.

### Whole Ferret Brain Datasets at Single-Cell Resolution

We used the MOST system to image the Nissl-stained brains and acquire whole-brain datasets for ferret at single-cell resolution. Ferrets are a non-primate model for studying the evolution and development of the mammalian cortex (Llinares-Benadero and Borrell, 2019) as well as perceptual information processing (Bimbard et al., 2018; Lempel and Nielsen, 2019). The ferret brains were used here to demonstrate the utility of our integrated pipeline for the construction and analysis of a cytoarchitectonic atlas of the large-volume mammalian brains than rodents.

**FIGURE 2:**
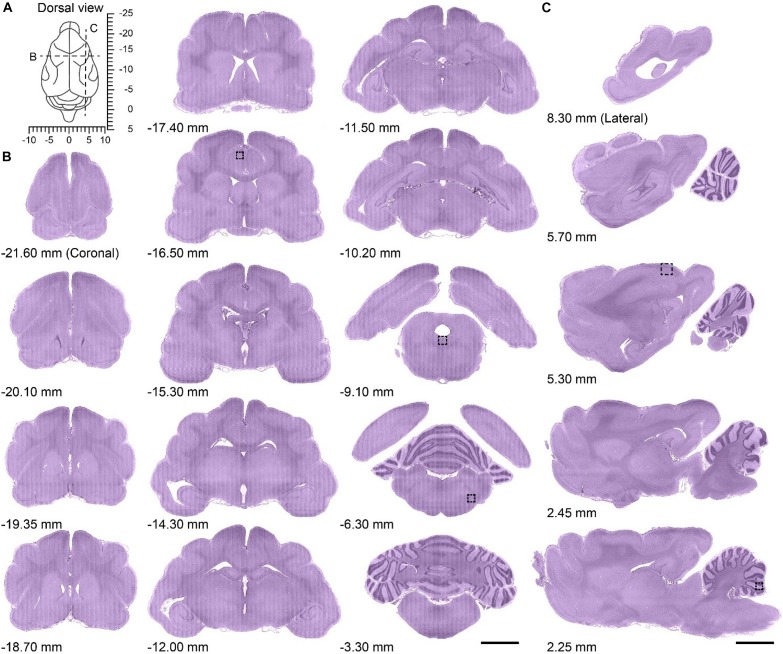
Serial coronal and sagittal slices from the whole ferret brain dataset. **(A)** Dorsal view of the ferret brain showing the anatomical location of serial coronal and sagittal planes along with stereotaxic coordinates (dotted lines in **B,C**); the occipital crest of the skull was used as the anterior-posterior origin (Radtke-Schuller, 2018). **(B)** Serial coronal slices of the whole ferret brain along with the approximate distance from the occipital crest line (first to third column from left to right; anterior side to posterior side, 20 μm thickness). Scale bar, 3 mm. **(C)** Serial sagittal slices of whole ferret brain along with approximate distance from the midline (fourth column from left to right; midline to lateral side, 20 μm thickness). Scale bar, 3 mm.

The brain was left intact and the 3D translation stage ensured accurate alignment of the images, allowing the acquisition of the whole-brain dataset at single-cell resolution without interruption or offset. The brain was imaged in the horizontal orientation, and could be re-sliced in the coronal and sagittal planes owing to the dataset characteristics of high resolution and continuity. The dorsal view of the ferret brain revealed the coordinate system taking the occipital crest of the skull as the anterior-posterior origin (Radtke-Schuller, 2018) as well as the location of the coronal or sagittal planes (Figure 2A). To assess the continuity of the dataset, a series of representative coronal slices from the olfactory bulb to the brainstem (Figure 2B) and sagittal slices from the lateral side to the midline (Figure 2C) were selected for analysis. The brain region contours were clearly distinguishable in all coronal or sagittal slices, which showed uniform staining.

**FIGURE 3:**
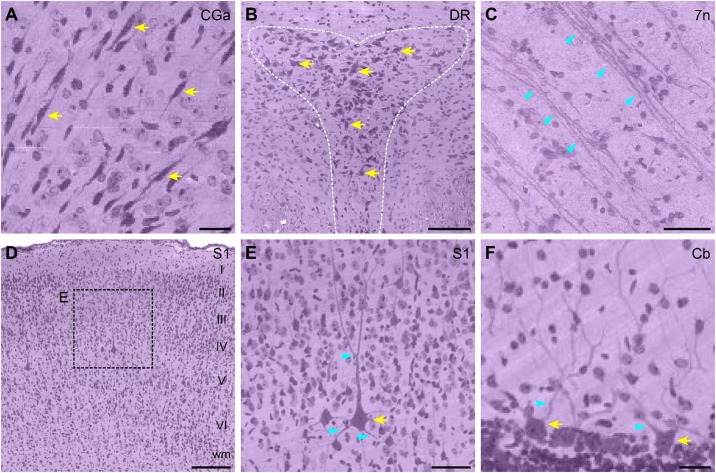
High-magnification view of single cell resolution images from the ferret brain dataset. **(A)** Enlarged view of bipolar cells in the CGa area. Scale bar, 20 μm. **(B)** Enlarged view of the DR nucleus (outlined with a white dotted line). Scale bar, 100 μm. **(C)** Enlarged view of fiber tracts in 7n. Scale bar, 40 μm. **(D)** Enlarged view of the cytoarchitecture of S1; layers I–VI can be clearly distinguished. Scale bar, 150 μm. **(E)** High magnification view of typical pyramidal neurons in S1, as shown in the dotted box in **(D)**. Scale bar, 50 μm. **(F)** Enlarged view of Purkinje cells in the 8th cerebellar lobule. Scale bar, 20 μm. All enlarged images are indicated by dotted boxes in Figures 2B,C. Yellow and cyan arrows indicate Nissl-stained cells and dendrites/fiber tracts, respectively. 7n, facial nerve; Cb, cerebellum; CGa, cingulate cortex, anterior part; DR, dorsal raphe; S1, primary somatosensory cortex; wm, white matter.

Enlarged partial images were selected to assess the resolution of the whole ferret brain dataset (Figure 3). The anatomical location corresponding to the images are shown in Figures 2B,C. The uniform staining throughout the brain enabled the delineation of contours of cortical structures such as the dorsal raphe nucleus (Figure 3B) and observation of layer-specific cytoarchitecture-for example, of the primary somatosensory cortex (Figure 3D). Various morphologically distinct cell types were identifiable including bipolar cells of the cingulate cortex (Figure 3A), pyramidal cells of the primary somatosensory cortex (Figure 3E), and Purkinje cells of the cerebellum (Figure 3F). Nissl-stained neurites or fiber tracts were also visible including the dendrites of pyramidal cells (Figure 3E) and Purkinje cells (Figure 3F) and fiber tracts of the facial nerve (Figure 3C).

### Giant PyNs in Ferret Brains

**FIGURE 4:**
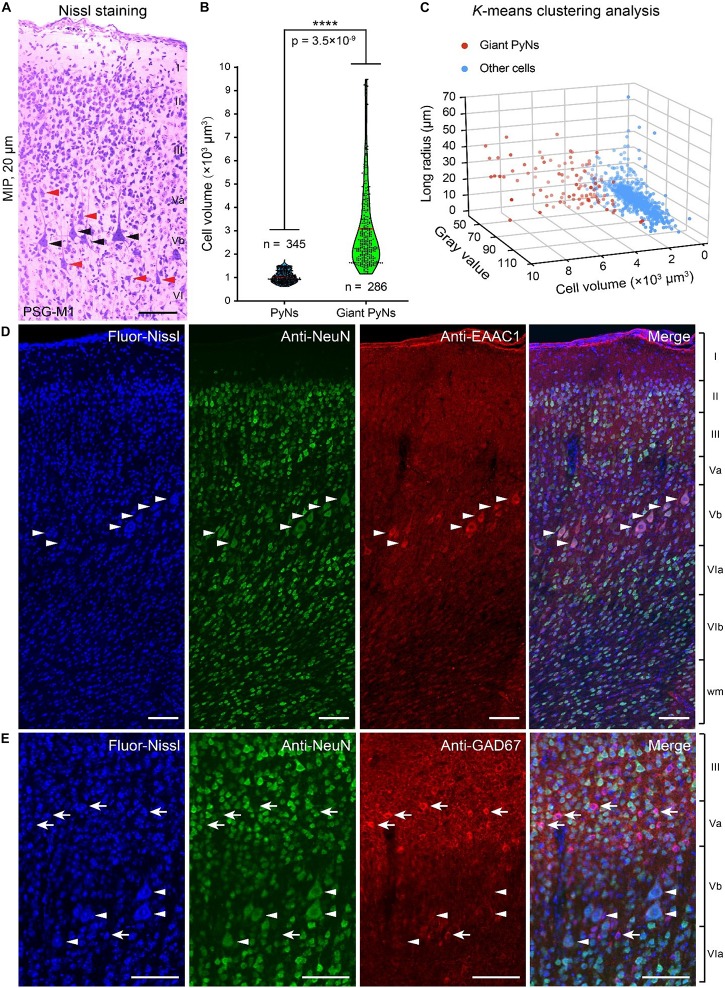
Giant PyNs in ferret primary motor cortex of the PSG. **(A)** Representative giant PyNs in primary motor cortex labeled by Nissl staining at 20-μm maximum intensity projection (MIP). Black and orange arrowheads indicate giant PyNs and other PyNs, respectively. Scale bar, 80 μm. **(B)** Cell volumes of giant PyNs and other PyNs in ferret motor cortex. Violin dot plots show the cell volume distribution of giant PyNs (*n* = 286 cells) and other PyNs (*n* = 345 cells). The width of colored regions represents density estimates; scattered points represent cell volume values; and the red line represents the mean cell volume of PyNs (95% confidence level, ****P < 0.0001; two-tailed Student’s *t* test). **(C)** 3D scatterplots of giant PyNs and other cell clusters based on the three principal components of cell volume, mean gray value, and longest radius (K-means clustering). **(D,E)** Giant PyNs were EAAC1-positive **(D)** and GAD67-negative **(E)** neurons. Blue fluorescent Nissl staining (first column) and anti-NeuN (second column), anti-EAAC1 **(D)**, and anti-GAD67 **(E)** immunolabeling were shown (third column) along with the merged images from the corresponding three channels (fourth column). White arrowheads in **(D,E)** indicated NeuN+/EAAC1+ and NeuN+/GAD67- PyNs, respectively; white arrows indicated NeuN+/GAD67+ neurons. Scale bar: 100 μm (**D,E**). Fluor-Nissl, Blue fluorescent Nissl; M1, primary or cortex; PSG, posterior sigmoid gyrus.

Giant PyNs (known as Betz cells) are a highly specialized subpopulation of giant pyramidal neurons in the motor cortex (Rivara et al., 2003; Kushchayev et al., 2012) that have been described in cat, dog, non-human primates (eg, chimpanzee, baboon, and monkey), and human (Rivara et al., 2003; Kushchayev et al., 2012; Barbas and Garcia-Cabezas, 2015). We identified giant PyNs in the ferret brain dataset. These cells had a larger soma and distinct dendrite morphology compared to typical PyNs (White et al., 1997; Rivara et al., 2003). The giant PyNs were distributed in layer Vb of the ferret motor cortex (Figure 4A).

In order to compare the giant PyNs to other PyNs of the ferret brain, we selected a set of PyNs (*n* = 631 cells, including 286 giant PyNs) from five data blocks (300 μm × 300 μm× 300 μm). Based on previously established criteria for identification of giant PyNs (White et al., 1997; Rivara et al., 2003), we performed manual soma segmentation in order to calculate cell volume, mean gray value, and longest radius. The mean cell volume of giant PyNs was 3101.31 ± 106.23 μm^3^ compared to 1004.32 ± 13.49 μm^3^ for other PyNs, representing a statistically significant difference (Figure 4B). We also carried out K-means clustering of manually segmented cells into 3D data blocks based on the three principal components of cell volume, mean gray value, and longest radius (Xu and Tian, 2015; Arora et al., 2016). These results showed that the giant PyNs clustered into two distinct subpopulations along with other cells (Figure 4C). The same result was obtained by K-means clustering into a single 3D data block (Supplementary Figures S2A–C).

To identify the neurochemical properties of giant PyNs, we selected another ferret brain and performed dual-immunolabeling for several molecular markers to characterize the neurochemical phenotypes of the giant PyNs in ferret brains (Figures 4D,E). One set of tissue sections were labeled with antibodies against NeuN (neuronal marker) and EAAC1 (pyramidal neuron marker, mainly expressing in pyramidal neurons), whereas another set of tissue sections were labeled with antibodies against NeuN and GAD67 (GABAergic neuron marker). All of the sections were counterstained with blue fluorescent Nissl dye. These results showed that the giant PyNs were NeuN+/EAAC1+ (Figure 4D) and NeuN+/GAD67- (Figure 4E), confirming their identity as excitatory pyramidal neurons (Conti et al., 1998; Zhou and Danbolt, 2013). As previously reported, most of Betz and layer 5 pyramidal neurons in several cortical areas of the macaque monkey weakly expressed the calcium binding protein parvalbumin (Ichinohe et al., 2004). To further verify whether the giant PyNs in ferret primary motor cortex expressed parvalbumin, we performed the immunolabeling against parvalbumin (PV) in ferret brain and confirmed that part of the giant PyNs in ferret primary motor cortex also were weakly PV-expression (Supplementary Figure S3).

### Brain-Wide Distribution of Giant PyNs

**FIGURE 5:**
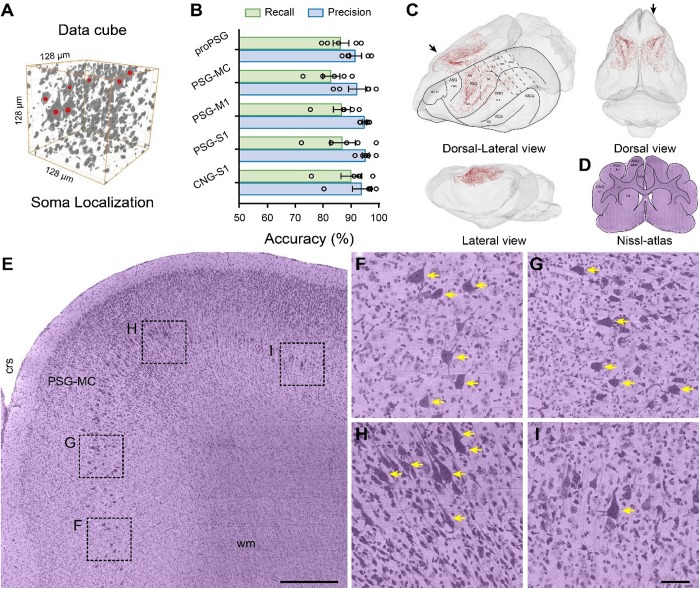
Brain-wide distribution of giant PyNs in whole ferret brains. **(A)** The location of giant PyNs within the 3D data cube was determined using the NeuroGPS algorithm; red dots indicate the center of somata of giant PyNs in the 128 μm × 128 μm × 128 μm 3D data cube. **(B)** Accuracy of automatic localization of giant PyNs (*n* = 5 data blocks [512 μm × 512 μm × 512 μm] per brain region). Green and blue columns represent the recall and precision values, respectively. **(C)** 3D visualization of brain-wide distribution of giant PyNs in various side views. Red dots indicate the center of somata of giant PyNs. Delineations of main cortical areas of ferret brain were plotted taking ferret brain atlas (Radtke-Schuller, 2018) and Nissl-cytoarchitectures as reference. **(D)** Delineations of brain areas of ferret brain in representative coronal plane, and the black arrow in **(C)** (dorsal-lateral view) shows the orientation of the coronal plane. **(E)** 2D view of giant PyNs distributed in the ferret motor cortex of the PSG (sagittal plane). The black arrow in **(C)** (dorsal view) shows the orientation of the sagittal plane. Scale bar, 400 μm. **(F–I)** High magnification views of giant PyN distribution, as shown in **(D)**. Yellow arrows indicate representative giant PyNs. Scale bar, 50 μm. AEG, anterior ectosylvian gyrus; ASG, anterior sigmoid gyrus; Cd, caudate nucleus; CGa, cingulate cortex, anterior part; CNG, coronal gyrus; crs, cruciate sulcus; cw, cerebral white matter; dPFC, dorsal prefrontal cortex; LG, lateral gyrus; M1, primary motor cortex; MC, motor cortex; MEG, medial ectosylvian gyrus; PMC, premotor cortex; PPc, posterior parietal cortex, caudal part; PPr, posterior parietal cortex, rostral part; PSG, posterior sigmoid gyrus; S1-3, primary/secondary/tertiary somatosensory cortex; wm, white matter.

The distribution patterns of giant PyNs were determined with the NeuroGPS algorithm (Quan et al., 2013). The somata of the giant PyNs in all TDat format 3D blocks throughout the brain were automatically located and counted using the L1 minimization model after foreground extraction. Only giant PyNs were analyzed according to predefined parameters of binarization and minimum radius (Figure 5A).

To evaluate the accuracy of the automatic giant PyN localization procedure, we randomly selected five data blocks in the pro-posterior sigmoid gyrus (proPSG), motor cortex of the PSG (PSG-MC), primary motor cortex of the PSG (PSG-M1), primary somatosensory cortex of PSG (PSG-S1), and primary somatosensory cortex of the coronal gyrus (CNG-S1) and compared the localization and quantification results obtained by automatic and manual methods. The highest and lowest recall values were 90.94 ± 3.84% and 83.58 ± 3.16%, respectively; highest and lowest precision values were 96.16 ± 1.26% and 92.49 ± 2.16%, respectively; and average recall and precision values were 87.36 ± 1.53% and 94.47 ± 1.03%, respectively. Three skilled investigators sequentially verified the automatic localization results to ensure reproducibility. The results demonstrate that the NeuroGPS algorithm can be used to automatically and accurately locate giant PyNs in the ferret brain (Figure 5B).

To determine the distribution of giant PyNs throughout the brain, we examined and showed the location of each of the cells from various side views (Figure 5C), and delineations of main cortical areas of ferret brain were plotted taking ferret brain atlas (Radtke-Schuller, 2018) and Nissl-cytoarchitectures (Figure 5D) as reference. The giant PyNs were mainly located in the motor cortex of the PSG, with sporadic distribution in some areas of the proPSG, PSG-S1, and CNG-S1. The total number of giant PyNs in the whole ferret brain was approximately 2.6 × 10^4^. The layer-specific distribution of giant PyNs in the motor cortex of the PSG was clearly visible in 2D (Figures 5E–I), and the large soma and intense staining of the giant PyNs distinguished them from other cell types (Figures 5F–I). This provides the first and most detailed description to date of the distribution of giant PyNs in ferret brain.

### Morphological Diversity of Giant PyNs

**FIGURE 6:**
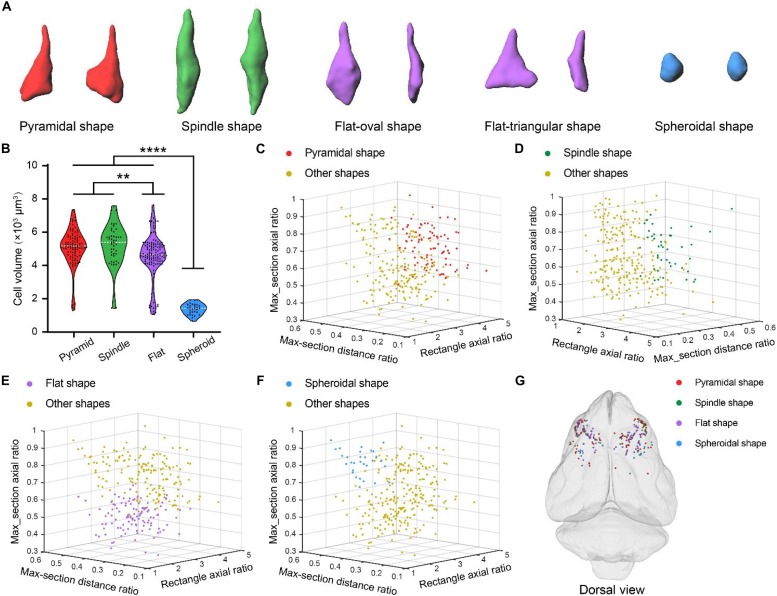
Diversity of Betz cell soma shape in ferret brain. **(A)** Surface rendering of four types of Betz cell soma shape including pyramidal, spindle, flat and spheroid. **(B)** Volumes of Betz cells with four types of soma shape. Violin dot plots represent the soma volume distribution of pyramidal (*n* = 93), spindle-shaped (*n* = 45), flat (*n* = 118), and spheroidal (*n* = 30) Betz cells. The width of colored regions represent density estimates; scattered points represent cell volumes; and the white line represents the mean cell volume of each soma shape type (95% confidence level, **P < 0.01, ****P < 0.0001; one-way analysis of variance followed by Tukey’s *post hoc* tests). **(C–F)** 3D scatterplots of four Betz cell soma shape types generated based on the three principal components of external rectangle axial ratio, maximum cross-sectional distance ratio, and maximum cross-sectional axial ratio (defined in Supplementary Figures S4B,C). **(G)** 3D visualization of brain-wide distribution of the four types of soma shape. 3D coordinates were recorded during automatic soma localization and dots with different colors represent the centers of the four soma shape types.

We next performed volumetric segmentation of the giant PyNs, hereafter referred to as Betz cells. To this end, we trained a 3D U-Net deep learning model-based convolutional neural network to automatically segment the somata of these cells (Ronneberger et al., 2015; Çiçek et al., 2016; Dong et al., 2018). Briefly, manually segmented Betz cells (*n* = 286) along with a data augmentation strategy were used to train the 3D U-Net model; the automatically located Betz cells were then segmented with the trained model, which showed excellent performance in the precise quantification of the cells (Figures 1C, 6A).

Betz cells in primates are heterogeneous in terms of soma size and shape (Hassiotis and Ashwell, 2003; Rivara et al., 2003). We also observed variability in the soma size and shape of Betz cells in ferret brain using the 3D U-Net deep learning model (Figure 6). The different types of soma shape were pyramidal, spindle, flat, and spheroid. The flat soma of Betz cells could be further classified as oval or triangular shaped (Figure 6A). To characterize the differences between the four types of Betz cell, we selected pyramidal (*n* = 93), spindle-shaped (*n* = 45), flat (*n* = 118), and spheroidal Betz cells (*n* = 30) for automatic segmentation and quantification. The mean cell volumes of the cells were 5065.95 ± 109.19 μm, 5223.36 ± 181.85 μm, 4551.30 ± 118.93 μm, and 1369.57 ± 59.2 μm, respectively. Thus, the rank order of Betz cells based on soma size is as follows: spindle-shaped > pyramidal > flat > spheroid (Figure 6B). We analyzed three soma parameters for the four types of Betz cell-namely, external rectangle axial ratio, maximum cross-sectional distance ratio, and maximum cross-sectional axial ratio (Supplementary Figure S4). The 3D scatterplots generated based on these parameters revealed that the four types of Betz cell could be easily distinguished from each other (Figures 6C–F) and had a mixed distribution pattern across the ferret brain (Figure 6G). These results demonstrate the phenotypic diversity of Betz cells in the ferret brain.

## Discussion

In this study we established an integrated pipeline for the construction of a whole-brain cytoarchitectonic atlas that involves uniform staining, whole-brain imaging, automatic soma segmentation, and quantitation of cells of interest at single-cell resolution in large-volume brains. We used the pipeline to generate a whole-brain cytoarchitectonic atlas for ferret and discovered specialized giant PyNs in ferret brains. We also mapped the distribution of these giant PyNs in the brain and demonstrated their diversity in terms of soma size and shape using big data analysis methods.

Information on brain cytoarchitecture is critical for investigating the organization and function of neural circuits, especially in the complex neocortex. Such information can be derived from Nissl or nuclear staining (Hezel et al., 2012; Wu et al., 2014; Seiriki et al., 2017); the former more readily stains the cell bodies and proximal dendrites of neurons (Dong, 2008; Van De Werd et al., 2010; Wu et al., 2014), whereas nucleic acid dyes mainly stain nuclei with weaker staining of the soma, which precludes the identification of landmarks in specific brain regions and cortical layers (Niu et al., 2015). However, existing Nissl staining protocols can only be applied to small brains such as that of rodents (Wu et al., 2014, 2016; Xiong et al., 2017); at present, there are no methods for effective en-bloc Nissl staining of large-volume brains.

To overcome the limitations of sample volume, we developed a modified large-volume en-bloc Nissl staining protocol for uniform staining of large-scale brain specimens. Intact brains with a volume greater than 6 cm^3^ such as that of ferrets were immersed in a concentrated (2.5%) faintly acidic (pH 5.0) thionine solution for up to 60 days, then thoroughly washed to prevent non-specific staining (Supplementary Figure S1). The constant environment as well as the long immersion time in the dye solution followed by extensive rinsing ensured adequate and uniform staining of the large brains. The subsequent resin embedding process allowed micron-level tissue sectioning for whole-brain imaging at single-cell resolution (Li et al., 2010; Wu et al., 2014). The MOST approach can be used to obtain a detailed 3D map of whole brains (Li et al., 2010; Wu et al., 2014; Yuan et al., 2015). Thus, by integrating the modified Nissl staining protocol with whole-brain imaging, it is possible to generate a whole-brain cytoarchitectonic atlas of large-scale brains at single-cell resolution and delineate different brain regions for quantitative analysis of brain structures of interest. It’s worth noting that the en-bloc Nissl staining results showed weak differentiation of the gray and white matter with different degrees of background staining, which may be caused by invalid alcohol differentiations. Thus, this en-bloc Nissl staining method cannot completely substitute traditional Nissl staining method performing on sections when high quality visualizations of cells are required. In addition, our integrated pipeline only performs single en-bloc Nissl staining although allows uniform true 3D reconstruction at cellular level. In contrast, the traditional neuro-histological method with MRI-guided registration allows multiple modalities (e.g., Nissl, Myelin and tract-tracing) to realize 3D reconstruction (Majka et al., 2016; Lin et al., 2019). Thus, the integrated pipeline is more suitable for construction cytoarchitectonic atlas at cellular resolution with single modality approach.

Ferrets are an ideal experimental animal model for studying cortical development, gyrification and perceptual information processing owing to their similarity to primates in terms of brain size and cortical organization, such as the brain volume expansion and the appearance of gyri and sulci than rodents (Rowell et al., 2010; Bimbard et al., 2018; Lempel and Nielsen, 2019; Llinares-Benadero and Borrell, 2019). However, to date there have been no true three-dimension cytoarchitectonic studies of the whole ferret brain other than the ferret atlas by Radtke-Schuller. Using our integrated pipeline, we obtained the first 3D whole-brain dataset for ferret brain at single-cell resolution. The cytoarchitecture of whole ferret brains were revealed by uniform staining with high contrast, from which we identified various cell types distinguished by morphology. Ferrets remain non-primate animal models, this pipeline may be applicable to other large-scale brains such as that of tree shrew (*Tupaia belangeri*), common marmoset (*Callithrix jacchus*) and even non-human primates.

Giant PyNs, also known as Betz cells (refers as giant pyramidal neurons locating in motor cortex in this study), are a highly specialized subpopulation of giant pyramidal neurons in the motor cortex with large cell bodies and unique dendrite morphology that have been described in cat, dog, non-human primates, and human (Rivara et al., 2003; Kushchayev et al., 2012; Barbas and Garcia-Cabezas, 2015). Here we report the existence of giant PyNs in ferret brain that we identified from the whole-brain ferret cytoarchitectonic datasets. We further identified the neurochemical properties of giant PyNs in ferret primary motor cortex as NeuN+/EAAC1+ (Figure 4D) and NeuN+/GAD67- (Figure 4E) with weakly PV-expression (Supplementary Figure S3). Previous study has shown that most of Betz and layer 5 pyramidal neurons in several cortical areas of the macaque monkey weakly expressed the calcium binding protein parvalbumin (Ichinohe et al., 2004). We also found that part of giant PyNs in ferret brain were weakly PV-expression with lower PV-expression than PV-positive interneurons (Supplementary Figure S3) in the present study. This discovery of giant PyNs and its neurochemical phenotypes characterization provide additional evidence for the similarity in brain organization between ferret and larger mammals.

To characterize the distribution of giant PyNs in the large-volume ferret brains, we employed a high-throughput data processing scheme consisting of the following steps: (i) TDat format transformation; (ii) automatic soma localization by NeuroGPS; (iii) 3D data block extraction based on 3D spatial coordinates; and (iv) automatic soma segmentation with a trained 3D U-Net deep learning model. The TDat platform reformats petabyte-scale whole-brain data into three-level 3D data (volume, cuboid, and block) and provides information on location in the data space and levels, which improves the efficiency of data reading and parallel computing (Li et al., 2017). We also determined the location of somata throughout the brain to obtain a more accurate picture of their distribution. For soma segmentation, a small 3D data block containing the cells of interest was extracted, which reduced data redundancy and improved data processing efficiency. Soma segmentation is typically performed using 3D projection images. However, advances in optical techniques have allowed the visualization of cell volume within large brains (Richardson and Lichtman, 2015; Susaki and Ueda, 2016). Deep learning is a useful approach for processing and analyzing 3D imaging data (Çiçek et al., 2016; Dong et al., 2018) that enables accurate volumetric segmentation in biomedical images (Ronneberger et al., 2015; Çiçek et al., 2016; Dong et al., 2018). In this study, we trained a newly 3D U-Net deep learning model for volumetric segmentation of the soma, which automated the process and provided accurate information on the location of individual cells in the ferret brain (Figures 1C, 6A). A noteworthy challenge is that how to correlate neuronal distribution patterns with precise brain area differences in three-dimensions, which is not solved in this study. In our vision, the precise 3D brain areas can be delineated by cytoarchitectonics or registration to MRI-atlas template. The pipeline includes the whole processes for en-bloc staining, acquisition of the information and analysis of the cellular characteristics and mainly applied to the characteristic analysis of giant PyNs with large soma size in present study. Currently, these soma segmentation methods were difficult to segment small or dense cells, which may be extended to quantitative analysis of other morphological cells with further optimizations of the soma segmentation methods, especially for small and dense cells.

## Conclusion

In conclusion, we propose an integrated pipeline for constructing a cytoarchitectonic atlas that involves uniform cell staining, whole-brain imaging, automatic soma segmentation using a deep learning model, and cell quantification at single-cell resolution in large-scale brains. The processing of dozens of terabyte-scale datasets was completed in 7 days. Using this approach, we identified giant PyNs with variable soma size and shape (pyramidal, spindle, flat, and spheroid) located in the motor cortex of the PSG in the ferret brains, and determined their brain-wide distribution patterns. This pipeline will be useful for mapping the cytoarchitectonic atlases of large-volume gyrencephalic animals and for comparative studies that can provide insight into the development and evolution of the neocortex.

## Data Availability Statement

All datasets generated for this study are included in the article/Supplementary Material.

## Ethics Statement

The animal study was reviewed and approved by the Institutional Animal Ethics Committee of Huazhong University of Science and Technology.

## Author Contributions

QL, HG, and XL conceived and designed the study. BL and SC developed the modified Nissl protocol and performed the staining as well immunolabeling experiments. TJ contributed to whole-brain data acquisition. AL, JZ, and XJ carried out the image preprocessing, TDat format transformation, and image re-slicing. AL, JZ, and XX modified the soma segmentation algorithm and determined the location of giant PyNs throughout the brain. BL analyzed the data and prepared the figures. BL, HG, and XL wrote the manuscript. All authors read the final manuscript and agreed to its submission.

## Conflict of Interest

The authors declare that the research was conducted in the absence of any commercial or financial relationships that could be construed as a potential conflict of interest.
