# Supplementary material for: Mapping the Architecture of Ferret Brains at Single-Cell Resolution
Source: Front Neurosci. 2020 Apr 15;14:322. doi: 10.3389/fnins.2020.00322 (PMC7174703; doi:10.3389/fnins.2020.00322)
Supplement: Supplementary file 1 [file Data_Sheet_1.docx]

**Supplementary figures**

**
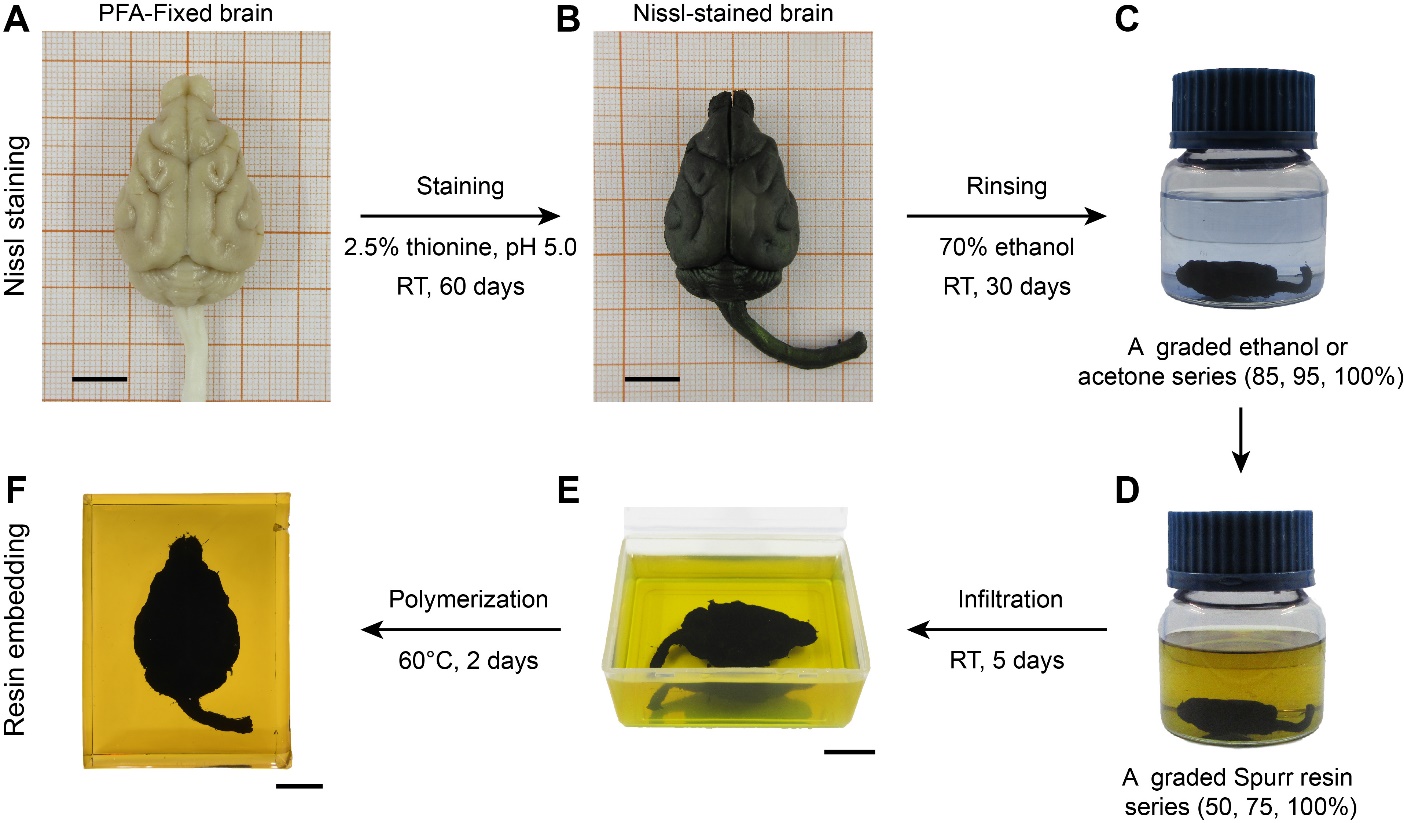
**

**Figure S1. Procedure for large-volume en-bloc Nissl staining and resin embedding for intact ferret brains. Related to Figure 1A.** (**A**) PFA-fixed intact ferret brain. (**B**) Nissl-stained intact ferret brain showing deep purple color. (**C**) Rinsing and dehydration through a graded ethanol/acetone series (70%, 85%, 95%, and 100%). (**D**) Resin infiltration through a graded Spurr resin series (50%, 75%, and 100%). (**E**) Polymerization of Nissl-stained intact ferret brain in a rectangular mold. The orientation of the brain was corrected as necessary. (**F**) Polymerized Nissl-stained intact ferret brain with an amber-like color. Scale bar, 1 cm.


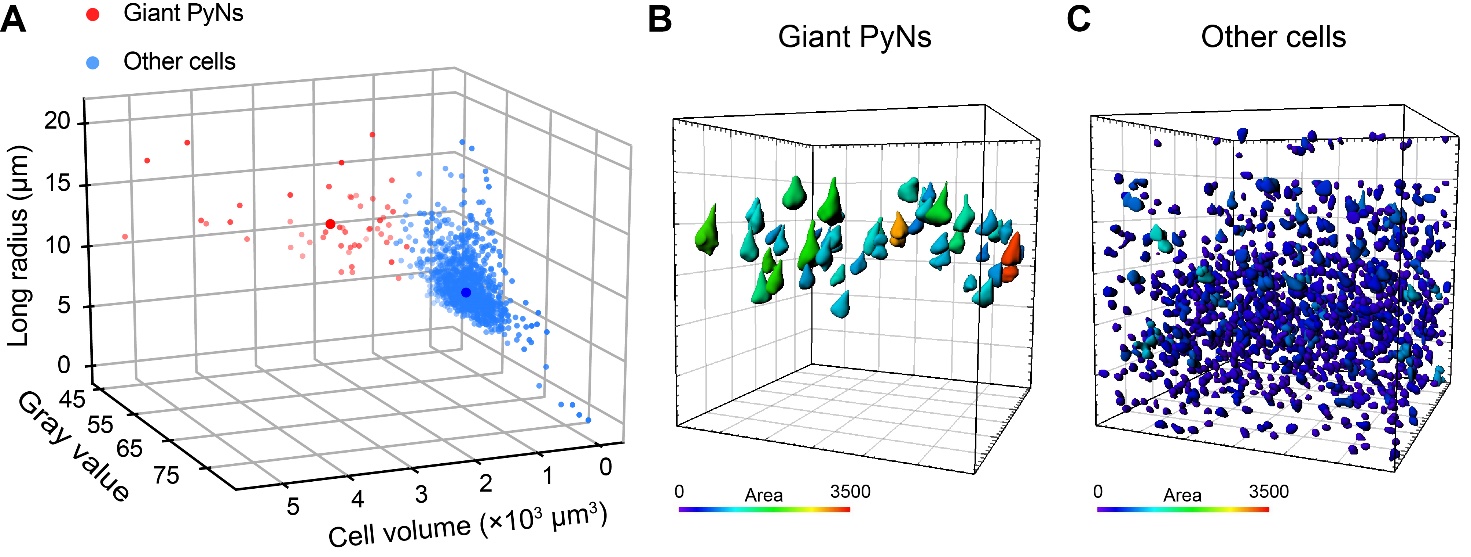


**Figure S2. Giant PyNs in the 3D data block of the ferret brain datasets. Related to Figure 4C.** (**A**) 3D scatterplots of giant PyNs and other cell clusters based on the three principal components of cell volume, mean gray value, and longest radius (K-means clustering). Large red dots and blue dots represent cluster centroids of giant PyNs and other cell types, respectively. (**B**) Surface rendering of clustered giant PyNs in the corresponding 3D data block. (**C**) Surface rendering of other cell clusters in the corresponding 3D data block. Imaris software was used for surface rendering, with a colored bar for a surface area of 0–3500 μm^2^ and 3D data block of 300 × 300 × 300 μm^3^.


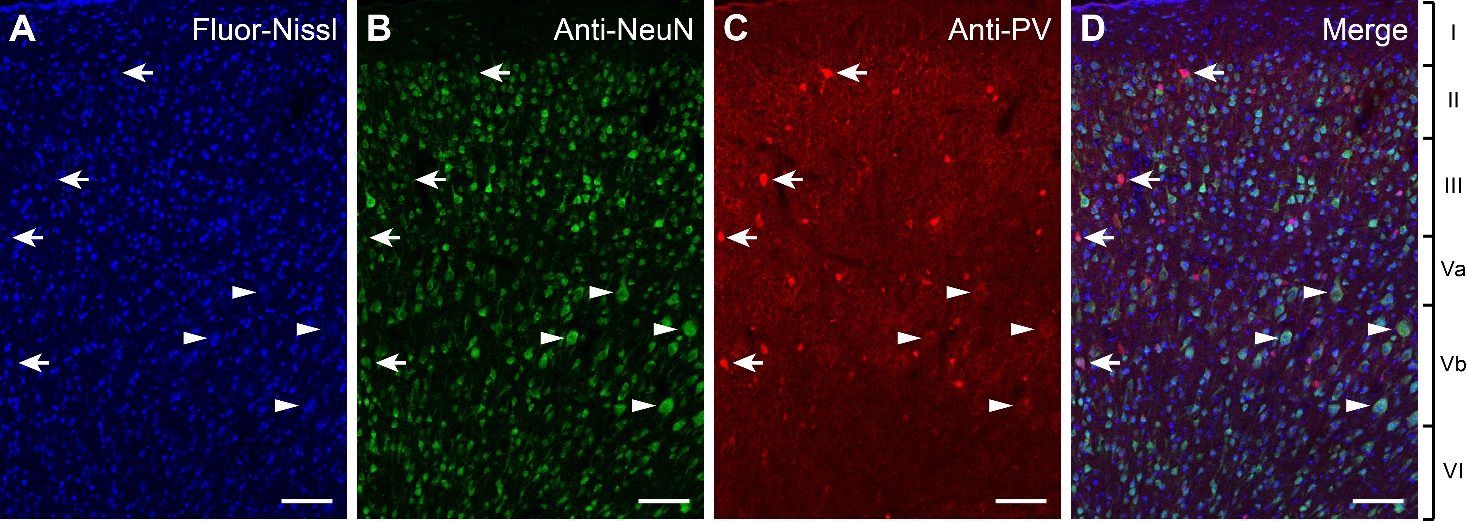


**Figure S3.** **Giant PyNs in ferret primary motor cortex were weakly PV-expression. Related to Figure 4D-E.** (**A-D**) Giant PyNs were NeuN-positive and weakly PV-expression neurons. Blue fluorescent Nissl staining (A), anti-NeuN (B) and anti-PV (C) immunolabeling were shown along with the merged images from the corresponding three channels (D). White arrowheads indicated NeuN-positive and weakly PV-expression PyNs and white arrows indicated PV-positive interneurons. Scale bar: 100 μm (A-D). Fluor-Nissl, Blue fluorescent Nissl; NeuN, neuronal nuclei; PV, parvalbumin.


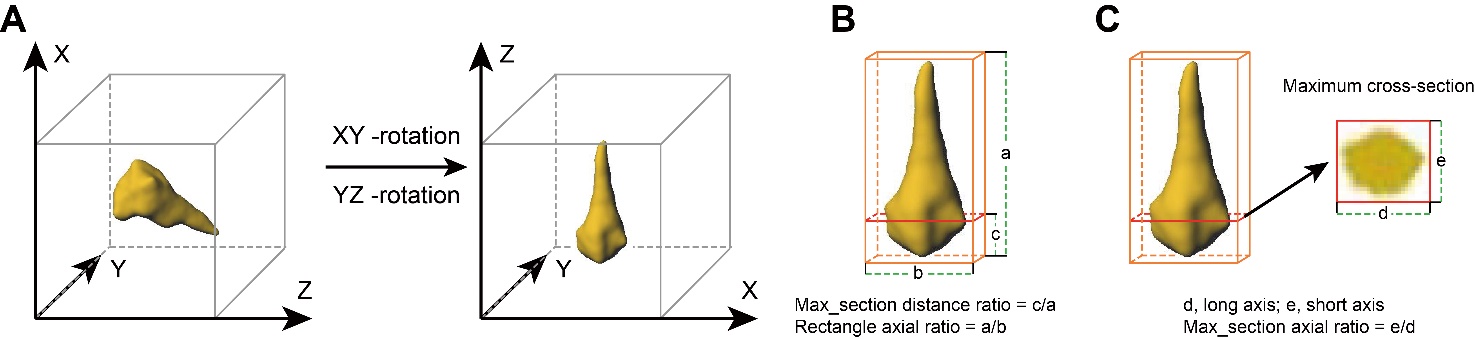


**Figure S4. Rotation and measurement of automatically segmented somata of Betz cells. Related to Figure 6C–F.** (**A**) Procedure for rotation and alignment of automatically segmented somata. The segmented soma was rotated and re-aligned in the XY and YZ planes (Wang et al., 2019). (**B**) Schematic of segmented soma parameters of maximum cross-sectional distance ratio (Max_section distance ratio) and external rectangle axial ratio (Rectangle axial ratio). (**C**) Schematic of segmented soma parameters of maximum cross-sectional axial ratio (Max_section axial ratio). **a**, Long axis of external rectangle; **b**, short axis of external rectangle; **c**, distance between maximum cross section and XY plane; **d**, long axis of maximum cross section; **e**, short axis of maximum cross-section.
